# The Worthy Person

**Published:** 1918-05-04

**Authors:** 


### HUMAN TEMPERAMENTS.

### THE WORTHY PERSON.

The Secretary walked up the path from the gate to the door of the hospital. His chin was in his right hand, his left hand supported his right elbow, and he looked on the ground, for he was thinking out a problem in finance. It was a cottage hospital, and the Secretary took great interest in every person and thing connected with it.

"Well, matron," he said, as she opened the door to him. "Well, matron," said he, raising his head, his eyes, and his voice, and rubbing his hands, for it was a cold day. "Well, matron," he said for the third time, "and how is the new nurse shaping?"

The matron met him cheerily, but a close observer might have thought her cheerfulness a little forced. "She is certainly very bright," said the matron, "and *most* willing and obliging."

The Secretary dropped his hands, his head, and his voice. "O Lord!" he groaned, "is it as bad as all that?" He had met worthy persons before.

The worthy person is always *most* willing and obliging, and is usually bright also. If a woman, she is very often bright, and proud of her brightness: it is her great asset. But whether bright or not, and sometimes he or she is lachrymose, he or she is always *most* willing and obliging. His favourite expression, or hers, as the case may be, is "Of course, if you wish it——" or "If I had only known you wished it!" If he has a defect, it is an inability to divine your wishes; but as soon as he knows them he is *most* anxious to carry them out.

You come in from a drive on a bitter winter's day, and you find the worthy person sitting reading by what was once a fire, but is now black out.

"Why on earth," you say, "did you let the fire out?"

"Oh," says the worthy person, "did you wish it kept in? If I had only known you wished it! Of course, if you wish it, I will keep it in in future." And she does. When the spring is far advanced and the birds are singing in the sunshine, when your winter clothing is becoming oppressive, and you mop your face as you come in from your walk, you find a great fire roaring up the chimney, and the room like a burning fiery furnace seven times heated.

"Good Lord!" you say, "you need not have made such a huge fire: it is quite a warm day."

"Oh," says the worthy person, brightly, "Didn't you wish it? I thought you always liked a big fire. You said, if you remember, that you liked a good fire kept up, but of course, if you wish it——" And next day when you come in, shrivelled, out of the bitter east wind, you find just a spark of fire in the grate, and the worthy person says, brightly, "You won't find the fire too much again. Now I know that you wish it, of course," etc.

The writer once employed a clerk to do some copying. He was a most worthy person, but like many worthy persons he was the butt of unmerited misfortune. His wife was ill, his fees were few, his child had been sent to an idiot asylum, he had lost his little savings in the Liberator smash. He was one of those persons

> whom unmerciful disaster
> Followed fast and followed faster, till his songs one burden bore,
> Till the dirges of his hope one melancholy burden bore, of "If you wish it: if I had only known that you wished it!"

His employer, a barrister, begged me to give the worthy man some remunerative employment for his leisure hours, and I was very willing to help a lame dog over a stile, but the difficulty was that there was nothing he could do. He was, so I was assured, a very competent barrister's clerk, but beyond clerking for a barrister he seemed to possess no accomplishment whatever. I was told, however, that he was a very careful and accurate copier, and I looked out some documents, and set him to copy them. I did not much want them copied, but I wanted to give the worthy person a leg up. The first document was a legal judgment several pages in length, that would take him, I felt assured, the greater part of the Saturday afternoon; so I set him to it, provided him with pens, ink, and paper, and went my way about my own avocations.

**HUMAN TEMPERAMENTS**—(*continued*).

At the end of an hour or so I visited him to see how he was getting on, for somehow I felt a misgiving: he was such a very worthy person. I found him sitting with the paper in front of him, gazing vacantly before him, with his hands in his lap.

"Well," I said, "can't you get on?"

"Oh, I have finished," said he. "I finished some time ago."

"Dear me, you must be a very rapid writer; let me see the copy."

He handed me a page and a half of manuscript, beautifully written in most legible script, but containing only a portion of the judgment, and leaving off in the middle of a sentence.

"This is very nice," said I. "Where is the rest of it?"

"That is all," said he. "There is no more." And he handed me the book.

I looked at it. "But this," I said, "goes down only to the end of the first page; where is the rest of it?"

"Oh, did you wish me to turn over the page? I did not understand that. Of course, if I had known that you wished it——"

The worthy man had ended his copying in the middle of a sentence, for he did not understand that I wished him to turn over the page. He was so worthy that, if he only had known, he would most certainly have turned it over. I am quite confident of that. However, he had one advantage that not every worthy person possesses. I could forgive him much, for at any rate he was not bright.

The worthy person is always willing and obliging. He does not mind how much trouble he takes as long as you are satisfied. He is always most anxious to please, and to save you trouble.

"What is it?" he says as you are rising from your chair. "Can't I do it? Let me get it for you."

"Well, thank you. I want Selden's 'Table-talk.' It is in the study, on the third shelf of the book-case on the right hand of the fireplace. About the middle."

He is gone an unconscionable time. You could have done it three times over in the time he takes; but presently he comes back.

"It was not where you said it was. It was on an upper shelf at the further end of the room. That was what kept me so long looking for it?" And he hands you Sheldon's "Fossils of the Upper Miocene."

After a morning's close application to your writing, you want to stretch your legs, and feel the need of a little exercise; so you go into the garden, take a hoe, and begin to massacre the weeds, an occupation you delight in. The worthy person is after you.

"Now, why will you tire yourself like that? Cannot the gardener do it? Let me fetch him. You will get your feet wet, and I am sure it is not good for you to stoop so much. Do let me fetch the gardener. You spoil that man, doing all his work for him. I am sure if we looked for him we should find him in the public-house. Well, then, let me get you a pair of goloshes. You like working in the garden? Then why not do some light work? Why not pick some flowers? I am sure there are plenty of roses. Look here, here's a beauty I have just found." And she shows you the bloom you have been nursing for the flower show, and hoped to take a prize with, now ruined for that purpose. She is such a worthy person, so willing, so obliging!

"Now that the garden is bursting and overflowing with vegetables of every description," you say to the worthy person, "I think we might have more variety at meals. Mashed potatoes round the mince, with boiled potatoes as the only accompaniment, is a little restricted, don't you think? Why not have some variety?"

"Oh certainly, if you wish it. There will not be the least difficulty about it. It is no trouble, but I did not know you wished it. You have only to say what you wish, and it shall be done. I will see that there is plenty of variety in future." So for the next week you have four vegetables with your meat every day, but—no potatoes. At length you ask if the scarcity of potatoes still continues.

"Scarcity?" says the worthy person. "Oh dear no! There are plenty. In fact we did not know what to do with all the gardener brought in, so we gave them away. I thought you did not like potatoes. You said, if you remember, that you wished—— I am most anxious to do everything you wish." And she is. She is a most worthy person.

The amiability, willingness, and obligingness of the worthy person would be tolerable if it were not for her brightness. She is proud of her brightness. She describes herself as bright, and she feels it incumbent on her to live up to her reputation for brightness; so she is all smiles. She is what used to be called "arch." She smiles archly as she tells you you are a terrible man. She is anxious you should not think she really means terrible, so she accompanies the adjective with an arch look and smile, to put you at your ease. If you are hard at work she thinks you are lonely and dull, and want brightening up, so she comes and talks to you, quite brightly. She feels you want brightening up, and she says so, and does her best to brighten you. She is such a worthy person.

The worthy man, to do him justice, is not often bright. On the contrary, he is usually a solemn, and sometimes a lachrymose person; but he is quite as willing and obliging as the worthy woman. He is most anxious to oblige. If he is going to town, he begs to be allowed to do your errands for you, and if you are incautious enough to entrust him with them, he takes immense trouble, and does not fail to let you know that he has done so. He spends a great deal of time in your service—he mentions it only to show you how willing he is to serve you—and he brings you with much display what you do not want and have no use for. He is such a worthy person.

